# Time-Course Lipidomics of Ornithine-Induced Severe Acute Pancreatitis Model Reveals the Free Fatty Acids Centered Lipids Dysregulation Characteristics

**DOI:** 10.3390/metabo13090993

**Published:** 2023-09-05

**Authors:** Jinxi Yang, Manjiangcuo Wang, Qi Qiu, Yan Huang, Yiqin Wang, Qianlun Pu, Na Jiang, Rui Wang, Li Wen, Xiaoying Zhang, Chenxia Han, Dan Du

**Affiliations:** 1 West China Centre of Excellence for Pancreatitis, Institute of Integrated Traditional Chinese and Western Medicine, West China Hospital/West China Medical School, Sichuan University, Chengdu 610041, China; yangjinxi9527@stu.scu.edu.cn (J.Y.); qiuqi@stu.scu.edu.cn (Q.Q.); huangyan@stu.scu.edu.cn (Y.H.); wangyiqin@wchscu.cn (Y.W.); stefzxy@gmail.com (X.Z.); hanchenxia@scu.edu.cn (C.H.); 2 Advanced Mass Spectrometry Center, Research Core Facility, Frontiers Science Center for Disease-Related Molecular Network, West China Hospital, Sichuan University, Chengdu 610041, China; wmjc@wchscu.cn (M.W.); puqianlun@wchscu.cn (Q.P.); jiangna1213@wchscu.cn (N.J.); wangrui5529@wchscu.cn (R.W.); 3 Peking Union Medical College Hospital, Chinese Academy of Medical Science & Peking Union Medical College, Beijing 100730, China; wenli@pumch.cn

**Keywords:** lipidomics, severe acute pancreatitis, free fatty acids, triglycerides, ceramides

## Abstract

The relationship between the type and intensities of lipids of blood and pancreas and the pathological changes in the pancreas during severe acute pancreatitis (SAP) remains unclear. In our study, we employed a rat model of SAP induced through intraperitoneal ornithine injections. We collected serum and pancreas samples at various time points (0–144 h) for histopathological and biochemical assessments, followed by lipidomic analyses using LC-MS/MS or in situ mass spectrometry imaging (MSI) To discern changes over time or at specific points, we employed time-course and univariate analyses for lipid screening, respectively. Our findings indicated that the peak inflammation in the Orn-SAP model occurred within the 24–30 h timeframe, with evident necrosis emerging from 24 h onwards, followed by regeneration starting at 48 h. Time-course analysis revealed an overall decrease in glycerophospholipids (PEs, PCs, LPEs, LPCs), while CEs exhibited an increase within the pancreas. Univariate analysis unveiled a significant reduction in serum TAGs containing 46–51 carbon atoms at 24 h, and CERs in the pancreas significantly increased at 30 h, compared with 0 h. Moreover, a substantial rise in TAGs containing 56–58 carbon atoms was observed at 144 h, both in serum and pancreas. MSI demonstrated the CERs containing saturated mono-acyl chains of 16 and 18 carbon atoms influenced pancreatic regeneration. Tracing the origin of FFAs hydrolyzed from pancreatic glycerophospholipids and serum TAGs during the early stages of inflammation, as well as FFAs utilized for CEs and CERs synthesis during the repair phase, may yield valuable strategies for diagnosing and managing SAP.

## 1. Introduction

Acute pancreatitis (AP) is an acute inflammatory disorder of the exocrine pancreas with no specific treatments [1]. Categorized by severity levels, the condition can be classified as mild acute pancreatitis (MAP), moderately severe acute pancreatitis (MSAP), and severe acute pancreatitis (SAP) [2]. Among these categories, SAP comprises 20%, often triggering systemic complications and multi-organ failure, with a mortality rate reaching up to 35% and a challenging prognosis [3,4]. To investigate the mechanism of this critical condition, one commonly employed rodent model is the noninvasive severe necrotizing AP model induced by intraperitoneal injection of high concentrations of cationic amino acids [5]. Studies have shown that three cationic amino acids, namely L-arginine, L-ornithine, and L-lysine, can induce severe necrotizing pancreatitis. Among these, the L-ornithine (Orn)-induced SAP model is considered the most reliable and severe, accurately reproducing the laboratory and morphologic characteristics observed in human pancreatitis [6,7]. Our recent study also points to the discovery of metabolic disorders associated with ornithine metabolism in SAP patients [8]. Although the Orn-SAP model has uncovered the pathological mechanism including activation of polyamine catabolism [9], blood–brain barrier damage [7] and mitochondrial injury [5], further studies are needed to fully describe this fatal disease.

In recent years, the metabolic disorder has become increasingly appreciated in the progression of AP [10]. As an important part of the metabolites, lipids have a wide range of biological functions, from providing energy storage and acting as a signaling molecule, to serving as structural components of membranes [11]. lipid homeostasis seems to be related to pancreatic metabolic balance [12]. Hypertriglyceridemia is the third most common cause of AP [13], and clinically, triglyceride-related metabolites are positively associated with the severity of AP [14,15] and pancreatic injury [16]. Lipotoxicity affects not only the multisystem organ failure of AP [17] but also regeneration after AP [18], and lipid saturation is also associated with lipotoxic systemic inflammation [19]; therefore, it is important to identify the lipids involved in the development of AP. We previously reported obviously changed phosphoglycerides and polyunsaturated fatty acyls are probably going to play an important role in the pathogenesis of a cerulein-induced AP mouse model [20]. However, the role of lipids in more severe models is far from clear.

Lipidomics is a method of study that focuses on the comprehensive analysis of lipid molecules within a biological system [21]. Lipidomics aims to provide a comprehensive understanding of lipid composition, distribution, and changes in different biological systems under normal and pathological conditions [22]. This detailed analysis of lipids can help uncover important insights into lipid metabolism, lipid-protein interactions, lipid signaling pathways, and their implications in various physiological and disease processes [23,24]. Currently, the study of lipidomics in AP is still in the early stage. Due to the inconsistency between peripheral circulation and local tissue, inferring correlated changes in situ is difficult. Therefore, simultaneous measurements of pancreatic tissues and serum lipidome can identify potential pathological indicators for mechanism study, related diagnosis and treatment. Here, we applied LC-MS/MS and mass spectrometry imaging (MSI) to detect serum and pancreatic tissue samples to screen for differential lipid metabolites in the Orn-SAP process. Dysregulated lipids were demonstrated by a broad-spectrum, targeted lipidomic profiling and the elucidation of lipid species interconversions conducted in both serum and pancreas. The results of the temporal lipidomics will give us new insight into biological processes and metabolic disturbances during SAP, and prompt possible interventions.

## 2. Materials and Methods

### 2.1. Animal Ethical Clearance, Acquisition, and Husbandry

Animal experimental procedures were approved by the Animal Ethics Committee of West China Hospital, Sichuan University (No. 20220519001). Male Sprague Dawley rats (220–230 g, 6–7 weeks) were purchased from Beijing Huafukang Bioscience Co., Ltd. (Beijing, China) and housed, up to four per cage, in a specific pathogen-free experimental animal center of West China Medical School on a 12 h light/12 h dark cycle at 25 °C. Rats were randomly grouped after one week of adaptive feeding. Food and water were supplied ad libitum.

### 2.2. Animal Experiments and Sample Collection

The model groups were established as our previously report [8] via the injection of Orn (2.8 g/kg body weight, 28%, i.p.) at hourly intervals for two times and sacrificed at the corresponding timepoint (6, 24, 30, 48, 72 and 144 h) from the first injection of Orn. The controls group (0 h) were treated with two injections of sanitary saline at 1 h intervals. All the animals were killed by an overdose of 5% isoflurane. For histopathologic evaluation, biochemical indexes and lipidomic analysis, 6–8 animals were used at each time point. After the pancreas was removed, it is divided into different parts, the parts used for histopathology was immediately fixed in 10% neutral formaldehyde, and the parts used for biochemical index and lipidomic analysis was snap-frozen and then stored at −80 °C until further processing. Blood was subjected to centrifugation at 1500× *g* for 15 min at room temperature, and the supernatant serum was collected and stored at −80 °C until processing. For MSI, 3 animals were used at each time point, and the entire pancreas was carefully removed, then immediately frozen and stored at −80 °C until further processing.

### 2.3. Histopathology and Biochemical Indexes Assessment

Histopathology. Fixed pancreatic tissue was embedded in paraffin and then sectioned into 3 μm slices for both H&E staining and immunohistochemistry targeting myeloperoxidase (MPO). In brief, for immunohistochemistry, tissue sections were deparaffinized and rehydrated, followed by microwave-based antigen retrieval under pH 6.0 conditions. After pre-blocking, the primary antibody (MPO, 1:200, A1374, ABclonal, Wuhan, China) was incubated overnight at 4 °C. Subsequently, the sections were incubated with a biotinylated secondary antibody, followed by 3,3′-Diaminobenzidine staining. Finally, the sections were counterstained with hematoxylin. The severity of pancreatitis was graded including measures for interstitial edema, leukocyte adherence and infiltration, vacuolization, apoptosis, necrosis, and regeneration [25,26], as shown in Table S1.

Biochemical assessment. Serum amylase and lipase activity were determined via α-amylase assay kit and lipase assay kit (C016-1-1, A054-2-1, Nanjing Jiancheng Bioengineering Institute, Nanjing, China) according to the manufacturer’s instructions. Pancreatic trypsin and MPO activity were measured according to previous study [27,28]. Serum alkaline phosphatase and alanine transaminase activity, urea and creatinine were assessed using Biochemical Analysis System (Cobas C702, Roche, Germany).

Total RNA was extracted from pancreatic tissue with TRIzol according to the manufacturer’s protocol. Total RNA (1 μg) was used for reverse transcription into cDNA in a total volume of 20 μL. All the steps were performed on ice-cold conditions. The relative expression of metabolic enzymes was detected via RT-qPCR. The primer sequences are listed in Table S2. cDNA (0.5 μL) was used as a template for PCR amplification by using an ChamQ SYBR color qPCR master mix (Vazyme Biotech; Nanjing, China) according to the manufacturer’s instructions. Relative expression of mRNA was calculated via the 2^−ΔΔCt^ method and Rpl13a ribosomal RNA was used for normalization.

All the statistical analyses were performed using GraphPad Prism (Version 9.0, GraphPad Software, Inc. San Diego, California, USA). The results were presented in mean ± SEM. A Student’s *t*-test was used to compare two groups, or one-way ANOVA analysis followed via Bonferroni post hoc test to compare multiple groups. A probability level of 0.05 was used as the threshold of significance.

### 2.4. Lipidomics Study

The preparation methods for rats’ serum and pancreatic tissue are consistent with our report previously [20], using a SCIEX Triple Quad^TM^ 5500+ mass spectrometer equipped with an LC-20AD UPLC system (Shimadzu, Kyoto, Japan) on an HSS T3 column (1.8 μm, 2.1 mm × 100 mm, Waters, Milford, MA, USA), controlled via Analyst software (Version 1.7.2, SCIEX, Framingham, MA, USA). SPLASH™ LIPIDOMIX^®^ Quantitative Mass Spec Internal Standard (330707-1EA, Avanti^®^ Polar Lipids, Alabaster, AL, USA.) used as internal standards (IS) according to the manual. A list of ion pairs detected in positive or negative mode can be found in Table S3.

The raw lipid data obtained from the measurements underwent several preprocessing steps. This included normalization with corresponding IS and quality control (QC) samples, as well as total strength normalization in both positive and negative ion modes. Lipids with missing values below 50% across all samples were selected for further analysis. The missing values of these lipids were estimated using the K Nearest Neighbor algorithm through the Wukong platform (https://www.omicsolution.com/wkomics/main/, accessed on 3 March 2023). Bioinformatics analyses in resulting processed data of lipid intensities were performed using the Wukong platform unless otherwise stated. Time-course analysis was employed to identify lipids exhibiting continuous fluctuations throughout the disease progression. Lipids with Hotelling T^2^ score greater than 30 were regarded as displaying continuous fluctuations over the disease course. These changed lipids were analyzed in principle components analysis (PCA), then principal component 1 vs. principal component 2 score of PCA scatter was used to depict metabolic trajectory. Simultaneously, a Mfuzz analysis was used to divide these changed lipids into clusters without prior specification, and proportions of each lipid subclass were accordingly calculated. The normalized data were combined and categorized into various lipid classes shown in the scatter diagram. Univariate analysis involved comparing specific time points to the baseline (0 h) using Student’s *t*-test, and the resulting *p* values were corrected using the False Discovery Rate method. A probability level of 0.05 was used as the threshold of significance. The fold change (FC) of lipids was calculated based on the mean of the respective groups. Variable Importance in Projection (VIP) scores were generated through Orthogonal Projections to Latent Structures-Discriminant Analysis (OPLS-DA) to assess the importance of metabolites. Data visualization was performed using the Wukong platform and GraphPad Prism 9.0.

### 2.5. Mass Spectrometry Imaging

The pancreatic tissues were sectioned to a thickness of 10 μm using a −20 °C cryostat microtome (CM 1950, Leica Microsystems, Wetzlar, Germany). These sections were then thaw-mounted on Superfrost Plus microscope slides (Thermo Scientific, Waltham, MA, USA). For MSI analysis, the tissue sections were allowed to dry in a vacuum desiccator for 3 h before analysis. Additionally, one of the consecutive tissue sections was utilized for H&E stain.

MSI were performed using an airflow-assisted desorption electrospray ionization (AFADESI) platform coupled to a Q-Orbitrap mass spectrometer (Q Exactive Plus Thermo Scientific, Waltham, MA, USA) as in our previous studies [29]. Mass data acquired by using Xcalibur software (Version 2.2, Thermo Scientific, Waltham, MA, USA). The original raw file data was converted to .cdf data file by using Xcalibur fileconvert function then input .cdf data into Massimager Pro (Version 1.0, Beijing, China) for tissue ion image reconstruction. After background ions subtraction, 6 regions on each tissue through a standard 3 × 3 pixel rectangle were selected, each region generated an independent mass profile containing m/z and intensity information as .txt format file. The respective data matrixes were then imported into the Markerview™ software 1.2.1 (AB SCIEX, Toronto, ON, Canada) for peak alignment and isotope peak removal, and then used for quantification. All the statistical analyses were performed using GraphPad Prism 9.0. The results of pixel rectangles data were presented in mean ± SEM. A Student’s *t*-test was used to compare two groups, or one-way ANOVA analysis followed via Bonferroni post hoc test to compare multiple groups. A probability level of 0.05 was used as the threshold of significance.

## 3. Results

### 3.1. Time-Course Biochemical Indexes and Pathological Changes of SAP Induced by L-Ornithine

**Figure 1:**
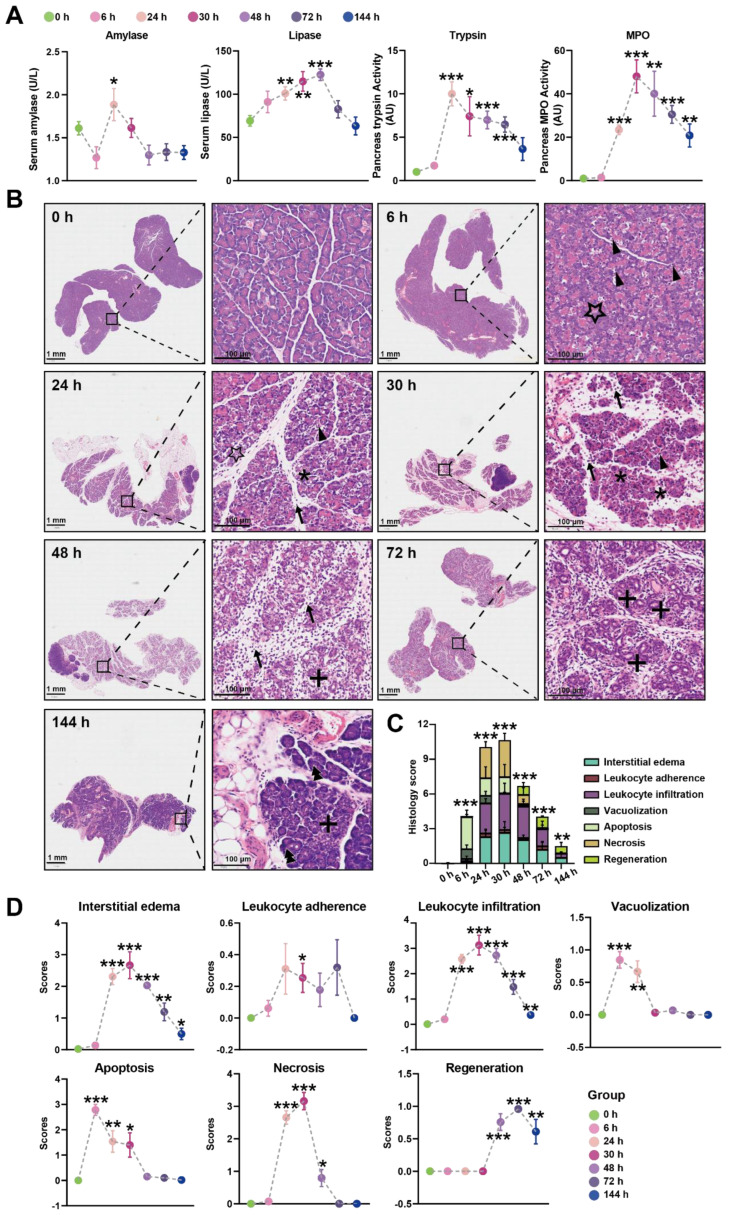
Time-course biochemical indexes and pathological changes of SAP induced by L-ornithine (2.8 g/kg × 2, 28%) (**A**) Multiple indicators of pancreatitis including serum amylase, serum lipase, pancreatic MPO, and pancreatic trypsin were assessed at 0, 6, 24, 30, 48, 72, and 144 h after the final injection of Orn. (**B**) Representative images of pancreatic histopathologic changes in response to intraperitoneal administration of Orn. Triangles for apoptotic bodies, pentagram for vacuolization, asterisks for necrosis, arrows for neutrophils, plus sign for ductuloacinar structures, and double triangle for the completed regeneration of acinar cells. Total histologic scores (**C**) and each histologic scoring (**D**) for pancreatic tissue samples in interstitial edema, leukocyte adherence and infiltration, vacuolization, apoptosis, necrosis, and regeneration at the different time points. * *p* < 0.05, ** *p* < 0.01, and *** *p* < 0.001 vs. 0 h; *n* = 6–8. Data are presented as mean value  ±  SEM.

In our initial experiments, intraperitoneal injection of Orn (2.8 g/kg × 2, 28%) in rats resulted in characteristic pancreatic necrosis and injury to remote organs, as depicted in Figure S1, based on these observations, this severe AP model was employed for the subsequent time-course study. All the measured parameters exhibited time-dependent changes, as illustrated in Figure 1A. Specifically, serum amylase activity reached its peak at 24 h and returned to normal levels by 48 h. Serum lipase activity continued to increase and reached its highest point at 72 h. Pancreas trypsin activity exhibited a rapid increase, peaking at 24 h, followed by a gradual decline. However, even at 144 h, the trypsin activity remained elevated compared to the baseline at 0 h. Pancreatic MPO activity reached its peak at 30 h, subsequently declining but still remaining higher than the levels observed at 0 h even at 144 h.

Histologic examination of the pancreas over time was performed using H&E images, revealing changes in the tissue (Figure 1B). At 6 h, pancreatic histopathology displayed a significant number of apoptotic bodies and foamy vacuolization of acinar cells. Interstitial edema became apparent at 24 h, accompanied by necrotizing acinar cells and the infiltration of neutrophils and monocytes in the interstitium. By 30 h, the inflammatory manifestations observed at 24 h persisted, with more severe edema, destruction of lobular structures, extensive necrosis of acinar cells, and increased infiltration of neutrophils and monocytes not only in the interstitium but also within the middle of necrotic lobules. At 48 h, the edema had attenuated compared to 30 h, and diffuse infiltration of neutrophils, monocytes, and macrophages was observed, gradually replacing the necrotic acinar cells with ductuloacinar structures. Inflammatory infiltration and significant collagen deposition in lobules and around ductuloacinar structures were still present at 72 h. At 144 h, atrophied ductuloacinar structures were observed, with newly formed acinar cells displaying zymogen granules, and a portion of the pancreatic parenchyma being replaced by fat. The total histologic score, displaying the overall severity, is shown in Figure 1C, while Figure 1D illustrates the specific pathological changes, including apoptosis, interstitial edema, leukocyte adherence and infiltration, vacuolization, and necrosis. In summary, combining the results of trypsin, MPO, and the histologic score, the peak of inflammation in the Orn-SAP model occurs at 24–30 h. The necrosis is obvious from 24 h. Over time, the necrotic pancreas begins to regenerate by 48 h, although the regeneration process is not complete by 144 h.

### 3.2. Time-Course Change in Lipids Perturbations in the Pancreas during Orn-SAP

**Figure 2:**
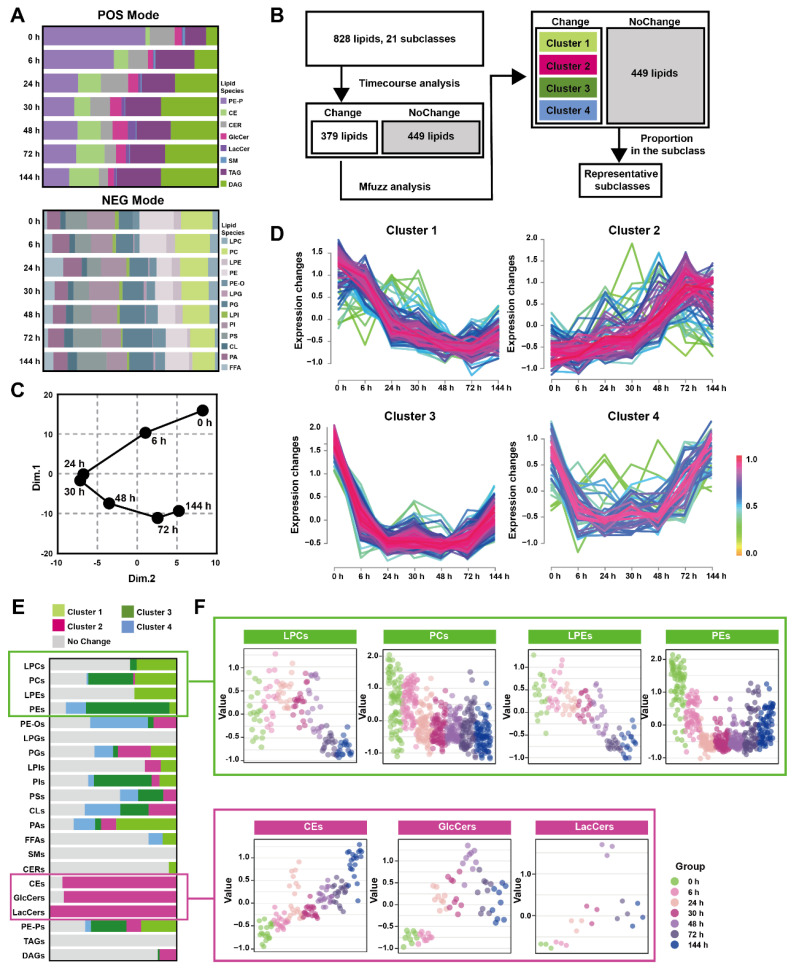
Time-course change in lipids perturbations in the pancreas during Orn-SAP (**A**) The stacked column charts indicated the composition of each lipid species detected in both positive (up) and negative (down) modes. (**B**) The analytical process for screening changed lipids over the Orn-SAP progression in the pancreas. (**C**) The principal component 1 vs. principal component 2 score trajectory plot showed the “open” trajectory status composed of mean points. (**D**) Mfuzz analyses divided the altered lipids in the pancreas into 4 clusters. (**E**) The proportion of lipid species enriched in each cluster in relation to the subclass to which they belong. Light green for cluster 1, rose for cluster 2, deep green for cluster 3, blue for cluster 4, and grey for no change. (**F**) Temporal lipids classified into corresponding subclasses (LPEs, PEs, LPCs, PCs, CEs, GlcCers, LacCers) shown by mean abundance in the scatter plots.

The pancreatic lipid extracts were subjected to broad-spectrum targeted lipidomic analysis in separate positive and negative modes, and 345 lipids from 8 different lipid subclasses were detected in positive mode and 483 lipids from 13 different lipid subclasses in negative. Figure 2A and Table S4 show the percentages of pancreatic lipid subclass abundance divided by the total intensity at multiple time points in two detection modes. The positive mode analysis revealed the presence of 43 1-(1Z-alkenyl),2-acylglycerophosphoethanolamines (PE-Ps), 20 cholesterol esters (CEs), 16 ceramides (CERs), 9 glucosylceramides (GlcCers), 3 lactosylceramides (LacCers), 1 sphingomyelin (SMs), 179 triacylglycerols (TAGs), 74 diacylglycerols (DAGs). On the other hand, the negative mode analysis detected19 lysophosphatidylcholines (LPCs), 79 phosphatidylcholines (PCs), 18 lysophosphatidylethanolamines (LPEs), 70 phosphatidylethanolamines (PEs), 22 hexadecyl-glycero-3-phosphoethanolamines (PE-Os), 4 lysophosphatidylglycerols (LPGs), 54 phosphatidylglycerols (PGs), 8 lysophosphatidylinositol (LPIs), 66 phosphatidylinositol (PIs), 56 phosphatidylserine (PSs), 18 cardiolipins (CLs), 42 phosphatidic acids (PAs), 27 free fatty acids (FFAs).

Figure 2B depicted the analytical process used to screening representative lipids related to the Orn-SAP procession in the pancreas. A total of 379 lipids with Hotelling T^2^ score greater than 30, determined through time-course analysis (Figure S2A) were screened for PCA analysis. The trajectory, represented by the average points of principal component 1 and principal component 2 (Figure 2C), showed an open state where the 144 h pancreatic lipid profile was close to but not fully recovered from the baseline at 0 h, while the profiles at 24 h and 30 h were distinct from the baseline. This lipid change trajectory is consistent with the peak time of pathological inflammation and necrosis as well as the result of incomplete repair at 144 h. Mfuzz analysis was conducted using 379 screened lipids, resulting in the grouping of lipids with similar trends into specific clusters (Figure 2D). Figure 2E shows the proportion of lipid species enriched in each cluster, categorized by their respective subclasses, with data detailed in Table S5. Most LPCs, PCs, LPEs, and PCs were enriched in cluster 1 and 3, denoted by light and deep green, respectively, exhibiting a decreasing trend. On the other hand, most CEs, GlcCers, and LacCers were enriched in cluster 2, indicated by rose color, and displayed an increasing trend. Figure 2F presents the temporal changes of lipid subclasses (LPCs, PCs, LPEs, PEs, CEs, GlcCers and LacCers), respectively. Notably, CEs exhibited an increase at all time points, while GlcCers and LacCers reached their peak at 72 h and subsequently decreased. Taken together, the time-course analysis of pancreatic lipidome indicated that three lipid classes-glycerophospholipids, CEs and ceramides-changed over time.

### 3.3. Lipids Changed at Certain Timepoints in the Pancreas during Orn-SAP

**Figure 3:**
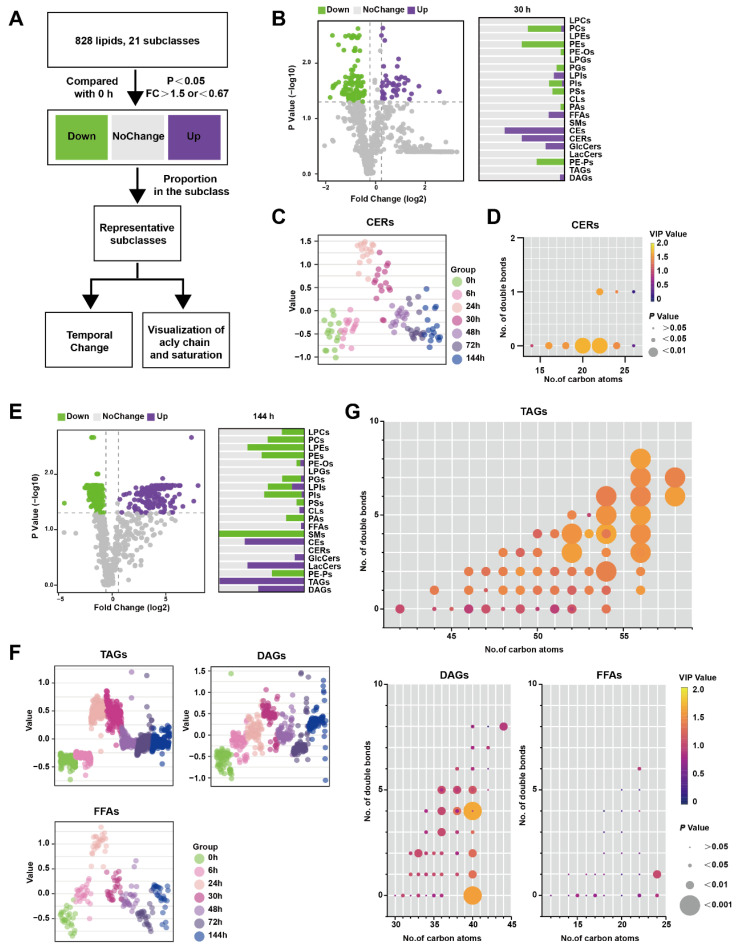
Lipids changed at certain timepoints in the pancreas during Orn-SAP (**A**) The analytical process for screening the lipids only changes significantly at a specific time point. (**B**) The volcano plots display the differential lipids between 30 h and 0 h. The x-axis represents the fold change (FC) of each lipid species, while the y-axis represents the statistical significance of the difference. Lipids meeting the significance threshold (*p* < 0.05) and FC criteria (>1.5 or <0.67) are indicated by purple circles (up-regulated), green circles (down-regulated), and gray circles (no change). The corresponding bar chart presents the proportion of up-regulated and down-regulated lipids within their respective subclasses. (**C**) Scatter plots depicted the temporal trend of CER lipid species changes. (**D**) The characteristics of conjugated double bonds and carbon atoms of CERs. The color brightness represents the VIP value, and the bubble size represents the *p* value. (**E**) The volcano plots display the differential lipids between 144 h and 0 h. and the bar chart shows the proportion of up- and down-regulated lipids to their subclasses. (**F**) Scatter plots revealed the temporal trend of TAG, DAG, and FFA lipid species changes. (**G**) Characteristics of conjugated double bonds and carbon atoms of TAGs, DAGs, and FFAs changed at 144 h. The color brightness represents the VIP value, and the bubble size represents the *p* value.

Figure 3A illustrates the analytical process used to identify lipids that exhibit significant changes at specific time points rather than over the entire time course. Lipids meeting the criteria of having *p*-values less than 0.05 and a FC greater than 1.5 compared to 0 h were classified as up-regulated, while lipids with P-values less than 0.05 and an FC less than 0.67 were classified as down-regulated. Lipids that did not meet these criteria were categorized as showing no change. In Figure 3B, the comparison between 30 h and 0 h is presented, depicting the proportion of various lipid species. Notably, CERs were found to be significantly up-regulated at 30 h. Figure 3C demonstrates that CERs reached their peak at 24 h and rapidly declined between 30 and 48 h. Furthermore, Figure 3D highlights that saturated CERs with 20–22 carbon atoms contribute the most to the up-regulation of this subclass.

Similarly, Figure 3E shows the results of the comparison between 144 h and 0 h, with the lipid species depicted as a proportion of their subclasses. TAGs were significantly up-regulated at 144 h, which was not observed in the previous time-course analysis (Figure 3E). TAGs reached their peak level at 24–30 h before DAGs but did not return to the baseline level at 144 h. FFAs, which are the hydrolysis products of TAGs and DAGs, exhibited a similar pattern, peaking at 24 h followed by a rapid decline (Figure 3F). To visualize the acyl chain characteristics of TAGs, DAGs, and FFAs at 144 h, the carbon atoms and double bond counts were analyzed. It was found that TAGs with more than 56 carbon atoms differed more from other TAGs, and the difference became more pronounced with an increased number of double bonds. (Figure 3G). Collectively, CERs only perturbed significantly at inflammatory peak time around 24–30 h, while both TAGs and FFAs increased at 24 h, and TAGs with long-chain unsaturated fatty acyls still maintained a high level at 144 h in comparison to 0 h. 

### 3.4. Time-Course Change in Lipids Perturbations in the Serum during Orn-SAP

**Figure 4:**
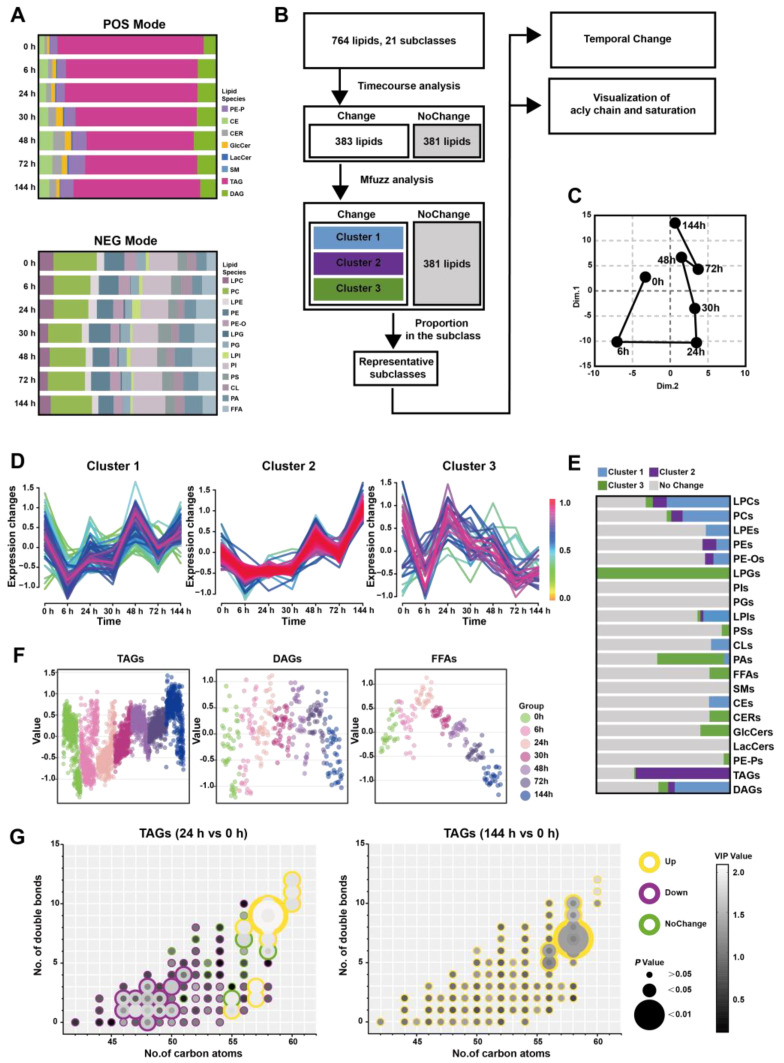
Time-course change in lipids perturbations in the serum during Orn-SAP (**A**) The stacked column charts indicated the composition of each lipid species in both positive (up) and negative (down) modes. (**B**) The analytical process for screening representative lipids related to the Orn-SAP procession in the pancreas. (**C**) The PCA score plot for time-point groups analyzed according to changed lipids from the time-course analysis showed the “open” trajectory status comprising mean points. (**D**) Mfuzz analyses divided the altered lipid in the serum into 3 clusters. (**E**) The proportion of lipid species enriched in each cluster in relation to the subclass to which they belong. Blue for cluster 1, purple for cluster 2, green for cluster 3, and grey for no change. (**F**) Scatter plots depicted the trend of TAGs, DAGs, and FFAs lipid species changes with time course. (**G**) The characteristics of conjugated double bonds and carbon atoms in fatty acyl chains of TAGs, DAGs, and FFAs in 24 h and in 144 h, compared with 0 h. Lipids meeting FC criteria (>1.5 or <0.67) are indicated by yellow circles (up-regulated), purple circles (down-regulated), and green circles (no change). The grayscale of bubbles represents VIP, while the size indicates the *p* value.

To further understand whether altered lipid metabolism in the pancreatic in situ is locally associated with the serum, we conducted a broad-spectrum targeted lipidomic analysis of multi-point serum from Orn-SAP in positive and negative modes. In the positive mode, we detected a total of 487 lipids belonging to 8 different lipid subclasses. These included 21 PE-Ps, 19 CEs, 13 CERs, 9 GlcCers, 2 LacCers, 1 SMs, 381 TAGs, and 41 DAGs. Similarly, in the negative mode, we identified 277 lipids from 13 different lipid subclasses. These subclasses consisted of 19 LPCs, 59 PCs, 11 LPEs, 29 PEs, 16 PE-Os, 1 LPGs, 13 PGs, 6 LPIs, 45 PIs, 16 PSs, 14 CLs, 22 PAs, and 26 FFAs (Figure 4A and Table S6).

Figure 4B demonstrates that the analytical process for screening changed representative lipids related to the Orn-SAP procession in the serum. 383 lipids with Hotelling T^2^ statistics greater than 30, based on time-course analysis, (Figure S2B) were screened for PCA analysis firstly. The trajectory plot, consisting of the average points of principal component 1 and principal component 2, showed an open state where the serum lipid profile at 144 h was close to but not fully recovered from 0 h, while 24 h time point was farthest from 0 h (Figure 4C). The temporal trajectory of lipids changes in serum were similar to that in pancreas. Next, Mfuzz analysis was done with those 383 lipids and the lipids with similar trends were grouped into the same cluster (Figure 4D). Figure 4E shows the proportion of lipid species enriched in each cluster in relation to the subclass to which they belong, with data detailed in Table S7. Notably, the majority of TAGs were enriched in cluster 2. Figure 4F shows the temporal changes of TAG, DAG, and FFAs, respectively. TAGs decreased at 24 h slightly and then increased, peaked at 144 h. DAGs showed no obvious change, while FFAs reached a peak at 24 h and then decreased, lower than 0 h at 144 h. To elucidate the characteristics of TAGs changes at 24 h and 144 h, we compared these two time points separately with the baseline (0 h). We then visualized the features including the number of carbon atoms, double bond count, VIP value, *P* value, as well as up- and down-regulated attributes (Figure 4G). At 24 h, TAGs with carbon atom counts of 46–51 were significantly downregulated, while TAGs with counts of 55–60 were significantly upregulated. At 144 h, TAGs with carbon atom counts of 56–58 were significantly upregulated. A commonality among these changes is that TAGs with a higher number of double bonds contributed more to the upregulated trend. In general, during the early stages of inflammation, the decrease changes in TAGs with carbon atom counts of 46–51 complement the trend of FFAs. On the other hand, the changes in TAGs with carbon atom counts of 56 or higher resemble those in the pancreas, especially at 144 h. 

### 3.5. FFAs-Centered Lipids Perturbations in the Pancreas during Orn-SAP

**Figure 5:**
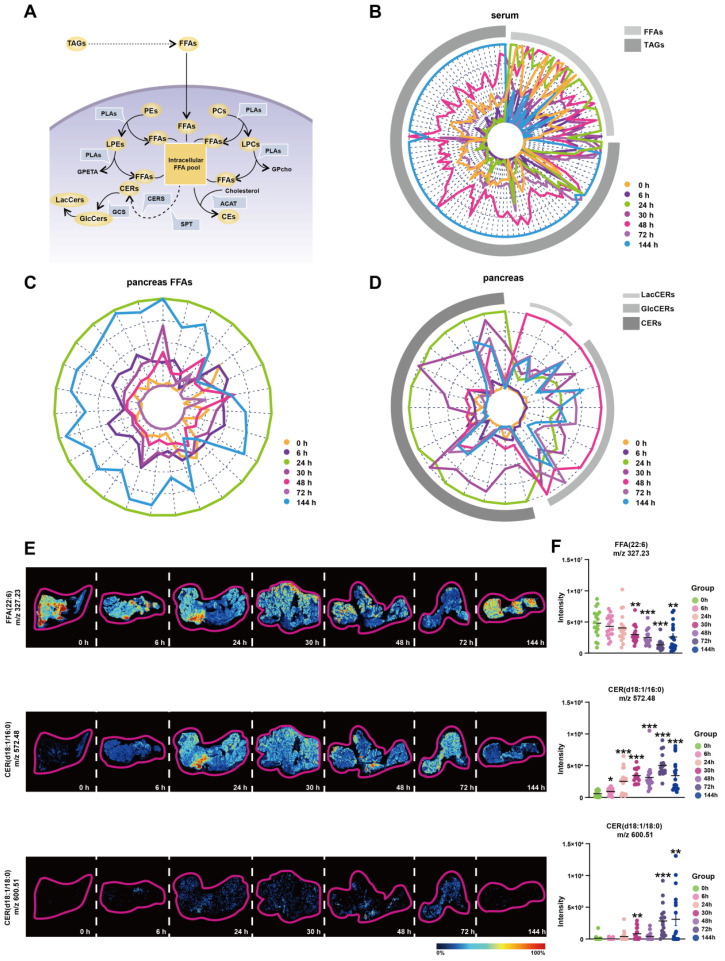
FFAs-centered lipids perturbations in the pancreas during Orn-SAP (**A**) Brief schematic diagram of FFA-related lipids metabolic pathways in acinar cells. The circles represent the lipid subclasses, the angular box represents the enzymes, and bold text without an outer frame indicates no detectable lipids. (**B**–**D**) Radar plots of FFA, TAG, CER, GlcCer, and LacCer changes in serum or pancreas at different time points (**E**) MSI of FFA (22:6), CER (d18:1/16:0), and CER (d18:1/18:0). The color bar from blue to red represents relative intensity. (**F**) The temporal MSI intensity of FFA (22:6), CER (d18:1/16:0), and CER (d18:1/18:0). * *p* < 0.05, ** *p* < 0.01, and *** *p* < 0.001 vs. 0 h. ACAT, acetyl-CoA acetyltransferase; GCS, glucose ceramide glucosyltransferase; CE, cholesteryl ester; CER, ceramide; CERS, ceramide synthase; FFA, free fatty acid; GlcCer, glucosylceramide; LacCer, lactosylceramide; LPC, lysophosphatidylcholine; LPE, lysophosphatidylethanolamine; MSI, mass spectrometry imaging; PC, phosphatidylcholine; PE, phosphatidylethanolamine; PLA, phospholipase A; SPT, serine palmitoyltransferase.

Based on the above analysis, we discovered the important changes of LPEs, PEs, LPCs, PCs, CEs, GlcCers, LacCers, and TAGs during Orn-SAP. These different lipid metabolites are interconnected with FFAs at the center (Figure 5A). The FFAs pool can be replenished by extracellular and intracellular pathways in pancreatic acinar cells. TAGs in pancreas and blood can be hydrolyzed to FFAs, and PEs, PCs, LPEs and LPCs are hydrolyzed in the presence of PLA to produce FFAs. The conversion of FFAs into lipid coenzyme A plays a crucial role in the de novo synthesis of CERs. This process involves the participation of metabolic enzymes such as serine palmitoyltransferase (SPT) and ceramide synthase (CERS). CERs can be converted to GlcCers and further to LacCers by glucosylceramide synthase (GCS). Cholesterol can bind FFAs in the presence of acetyl-CoA acetyltransferase (ACAT) to produce CEs. Overall, this interconnected network of lipid metabolites, with FFAs as a central component, highlights the dynamic processes and transformations involved in Orn-SAP.

To enhance our understanding of the transformation relationships among these lipids at various time points, we have depicted their lipid content levels using radial plots, depicting changes of TAGs and FFAs in serum; changes of FFAs in pancreas; and changes of CERs, GlcCers, and LacCers in pancreas, respectively. In these plots, each line corresponds to a specific lipid, while arcs in various shades of grey represent different lipid subclasses. We have employed distinct colors to differentiate between the different time points, allowing for easier interpretation of the transformation patterns. Figure 5B shows the changes of TAGs and FFAs in the serum. The green curve, representing 24 h, exhibits an expansion of the FFAs arcs, indicating an increase in FFAs. Conversely, the blue curve, representing 144 h, demonstrates an expansion of the TAGs arcs, suggesting an increase in TAGs. These findings reveal distinct shifts trends in the levels of FFAs and TAGs over time in the serum. Figure 5C shows the changes of FFAs in the pancreas, The outermost circle, represented by green, corresponds to 24 h, and as the time progresses, the circle gradually shrinks, indicating a decrease in FFAs. However, the circle corresponding to 144 h, denoted by blue, expands, suggesting an accumulation of FFAs at this time point in the pancreas. Figure 5D shows the changes of CERs, GlcCers and LacCers in the pancreas. The curve associated with 24 h, represented by green, expands under the CERs arcs, indicating an increase in CERs. Conversely, the curve representing 48 h, indicated by red, exhibits a reduction in the CERs arcs while expanding under the LacCers and GlcCers arcs. These complementary curves form a cohesive circle, suggesting an intrinsic link in the transformation of lipids between CERs, LacCers, and GlcCers. The mRNA expression of relative metabolic enzymes, including SPTC1, SPTC2, CERS2, CERS4, ACAT2, and GCS, were determined in pancreas, which supported the possible synthesis of CEs, and inter-conversion among CERs (Figure S3).

To validate the findings of lipidomics analysis, we combined untargeted AFADESI-MSI to extract representative images of entire pancreatic sections. Representative lipids FFA (22:6) and CER (d18:1/16:0) and CER (d18:1/18:0) images with the same overall trend of FFAs and CERs lipid groups were shown in Figure 5E. Six regions on each tissue through a standard 3 × 3 pixel rectangle were selected, each regions generated an independent mass profile containing m/z and intensity information, which was shown in Figure 5F.

Overall, these results suggest that there is a critical interconversion between serum and pancreas for FFAs, TAGs, CERs, GlcCers and LacCers during Orn-SAP and that it is closely linked to the progression of Orn-SAP.

### 3.6. Histopathological Heterogeneity Findings Correlated with In Situ Lipid Levels of CERs at 144 h of SAP

**Figure 6:**
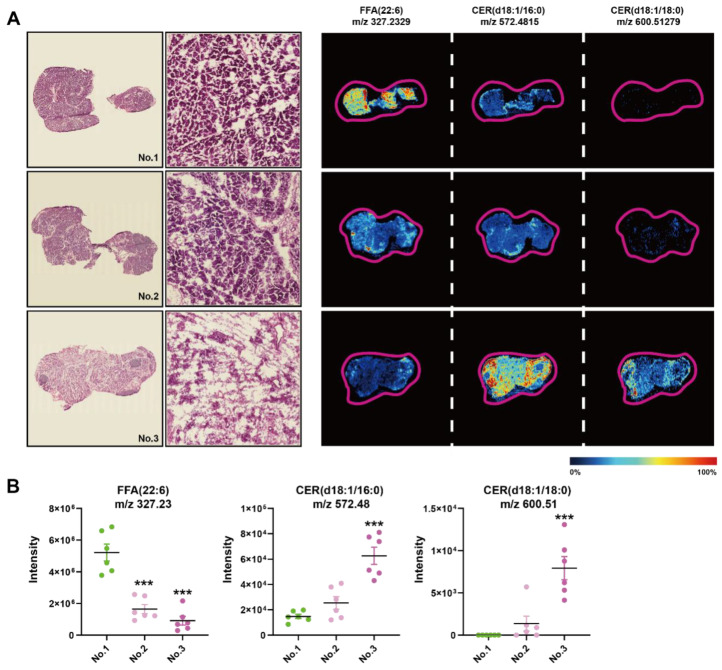
Histopathological heterogeneity findings correlated with in situ lipid levels of CERs at 144 h of SAP (**A**) H&E staining images of the frozen sections of pancreas at 144 h and corresponding MSI of FFA (22:6), CER (d18:1/16:0), and CER (d18:1/18:0). The color bar from blue to red represents relative intensity. (**B**) The MSI intensity of FFA (22:6), CER (d18:1/16:0), and CER (d18:1/18:0) at 144 h. *** *p* < 0.001 vs. 0 h. *n* = 6 religions. Data are presented as mean value  ±  SEM.

We found a large intergroup heterogeneity at 144 h during the processing of AFADESI-MSI data; therefore, we examined the HE images and MSIs of FFAs and CERs of the consecutive slices from the same sample at 144 h (Figure 6A). There were more completed regenerated acinar cells in the first sample compared to the second, of which the ductal structure was still visible, while the third one exhibited few intact acinar cells. In the first sample, the intensity of FFA (22:6) was significantly higher compared to the other two samples, and the intensity of CER (d18:1/16:0) and CER (d18:1/18:0) was the opposite, especially compared to the third (Figure 6B). Therefore, CERs and FFAs concentrations had a greater impact on the repair results of the Orn-SAP model maintained a high level at 144 h in comparison to 0 h.

## 4. Discussion

We induced SAP in rats by administering ornithine via intraperitoneal injection. This model resulted in extensive pancreatic parenchyma necrosis, along with noticeable impairment in distal organs, notably the lung, kidney, and liver (Figure S1). Previous research conducted by Biczo et al. demonstrated impaired lipid metabolism in a model of arginine-induced AP [30]. Given that Orn is a byproduct of arginine metabolism in vivo and has been shown to induce more severe pancreatic injury compared to arginine [26], we chose to establish the ornithine-induced SAP model in order to investigate the associated lipid changes. For consistency, we utilized the equimolar dose of arginine as a reference point for our experiments.

The pathogenesis of TAGs as a causative agent of AP is not clear, and elevated TAGs in AP progression have been demonstrated [13]. One of the main current theories for its exacerbation of AP is the release of excess FFAs from hydrolyzed TAGs via lipases [31], which is often described as a major lipotoxic player in AP. Unsaturated FFAs have been reported to cause pathological intracellular calcium elevation, inhibition of mitochondrial complexes, and promotion of necrosis in pancreatic acinar cells [17]. In addition, saturated FFAs can cause ER stress [30]. but so far, the relevance of TAGs and FFAs changes in serum and pancreas in Orn-induced SAP has not been elucidated. The mobilization of serum TAGs with 46–51 carbon atoms may be related to the increased lipase activity released into the blood after pancreatic necrosis, which corresponded to the rapid elevation of FFAs, the hydrolysis product of TAGs, in the serum. TAGs in the pancreas increased from 0 to 24 h, which may be related to the progressive loss of exocrine function of the pancreas and the formation of lipid droplets; however, the level of FFAs in the pancreas increased instead at this time. Large autophagic vacuoles containing zymogen particles appeared at 6 h, and lipid droplets persisted in the basal part of the acinar cells during the first 24 h of the disease course. Our previous pancreatic proteomic analysis of the cerulein-induced model [20] suggests that phospholipases appear to be heavily expressed in the early stages of inflammation. In our results, the abundance of PEs and PCs, important components of biofilms, continued to decrease, indicating their degradation likely involved in autophagic processes in acini. Therefore, we hypothesized that the dramatic increase in FFAs in the pancreas during the first 24 h might be mainly phospholipid hydrolysis and serum FFAs transport rather than pancreatic TAGs hydrolysis in situ.

Both serum and pancreatic FFAs decreased rapidly after 24 h, suggesting a significant use of it by the pancreas. This utilization was delayed, and occurred after inflammation. We found a significant upregulation of TAG whole family abundance in the pancreas at 144 h, and similar results were obtained from the analysis of serum lipidomics. Further analysis of TAG lipid profile in the pancreas at 144 h revealed that the accumulation was mainly of TAGs containing highly unsaturated long-chain fatty acyl groups, and such higher unsaturated TAGs containing more than 56 carbon atoms remained above the 0 h state throughout the time course. In contrast, FFAs in the pancreas continued to decrease after 24 h and showed a state close to 0 h during 144 h recoveries, a process in which the contribution of unsaturation and carbon chains to the course of the disease did not differ significantly, hinting that a sustained mobilization of all FFAs in the late phase of inflammation.

As a precursor for most lipid synthesis, FFAs can bind free cholesterols in the presence of ACAT to produce CEs [32], which was supported by their persistent increase in the pancreas. Additionally, the change in CERs, GlcCers, and LacCers is obvious. As a downstream product of CERs, GlcCers and LacCers increased rapidly at 24 h and peaked at 48 h. The changes in CERs showed a bit more complexity. It started to increase at 6 h, peaked at 24 h, and declined rapidly at 48 h. We used radar plots to manifest this interconnected change in CERs, GlcCers, and LacCers. The two complementary curves of 24 h and 48 h was shown a substantial conversion of CERs to GlcCers and LacCers during this period (Figure 5D). The classical understanding of CERs is their effect on apoptosis [33], thus elevated CERs may be directly related to the appearance of apoptotic vesicles starting at 6 h in the pancreas. Moreover, GlcCers has been proven to activate hepatic stellate cells in liver fibrosis [34]. Pancreatic stellate cells are homologous to hepatic stellate cells and are involved in early regeneration after pancreatic parenchyma destruction [35]. Consequently, we speculate that the increased GlcCers in the pancreas may be involved in the anti-inflammatory and repair processes and that its excessive accumulation could potentially be one of the reasons for the shift in AP prognosis to chronic pancreatitis.

There was large heterogeneity in the pathological findings at 144 h. Some had developed complete acinar structures and ductal degeneration, while others were still in the process of ductal differentiation. By comparing this histopathological difference, we hoped to further contrast the discovery of metabolite associations with cell type and function. Combined with the MSI results of the pancreas, we found that CERs correlated with the degree of pancreatic regeneration. It has been reported that the lipotoxicity of CERs was related to their acyl chain length and saturation or not [36,37]. CERs with saturated mono-acyl chain of 16 and 18 carbon atoms have more intense lipotoxicity [37], which was in accordance with our results that the abundance of these CER subspecies was significantly lower in the well-regenerated pancreas than in the poorly regenerated samples.

Finally, a few limitations of this study need to be mentioned. Firstly, it is important to note that the lipidomics in this study provides results in relative quantification. Secondly, the coverage and resolution limitations of AFADESI-MSI restrict our ability to observe correlations between a broader range of lipid types and finer pathological changes. Thirdly, 144 h may not be a long enough time point to observe the end of the Orn-SAP model. Lastly, due to the multi-organ damage associated with SAP, changes in the serum lipids could stem from various sources, particularly the liver. In future research, integrating multi-organ omics results could aid in gaining a better understanding of the unique progression of SAP.

## 5. Conclusions

In summary, our lipidomic analysis indicated that around the inflammatory peak phase with higher pancreatic enzyme activities, the rapid increase in pancreatic FFAs is primarily attributed to the massive hydrolysis of in situ PEs and PCs, rather than TAGs, and TAGs mobilized from the peripheral circulation During the pancreatic regeneration phase, there is a predominant consumption of FFAs, which is closely associated with the production of CERs, CEs, and TAGs. The progression of pancreatic regeneration is related to TAGs with greater unsaturation levels and carbon atoms in acyl chains, as well as CER (d18:1/16:0) and CER (d18:1/18:0) species. Hence, the sources of FFAs in the pancreas and circulation are different during the peak phase of inflammation, and the unsaturation and carbon chain length of FFAs may affect the repair of the pancreas, which provides important ideas for disease diagnosis and treatment.

## Data Availability

The data supporting the results of the study can be found within the article and the supplementary material.

## Supplementary Materials

The following supporting information can be downloaded at: https://www.mdpi.com/article/10.3390/metabo13090993/s1, Figure S1. Pancreatic and distal organ damage in the model of ornithine-induced severe acute pancreatitis (2.8 g/kg × 2, 28%); Figure S2. The differential lipids screened by time-course analysis and shown in a volcano plot; Figure S3. The relative pancreatic mRNA expression of enzymes relevant to the lipid in Orn-SAP; Table S1. Histological scoring system for the evaluation of pancreatic injury in rats; Table S2. Sequence of RT-qPCR primers; Table S3. Ion pairs detected in positive and negative model .xlsx; Table S4. Lipids in pancreas .xlsx; Table S5. Counts and proportion in pancreas .xlsx; Table S6. Lipids in serum .xlsx; Table S7. Counts and proportion in serum .xlsx.
