# Supplementary material for: Time-Course Lipidomics of Ornithine-Induced Severe Acute Pancreatitis Model Reveals the Free Fatty Acids Centered Lipids Dysregulation Characteristics
Source: Metabolites. 2023 Sep 5;13(9):993. doi: 10.3390/metabo13090993 (PMC10647642; doi:10.3390/metabo13090993)
Supplement: Supplementary file 1 [file metabolites-13-00993-s001.zip › Supporting Information.pdf]

Supplementary Materials for

Time-course Lipidomics of Ornithine-induced Severe Acute Pancreatitis Model Reveals the Free Fatty Acids Centered Lipids Dysregulation Characteristics

Jinxi Yang[1#], Manjiangcuo Wang[2#], Qi Qiu[1], Yan Huang[1], Yiqin Wang[1], Qianlun Pu[2], Na Jiang[2], Rui Wang[2], Li Wen[3], Xiaoying Zhang[1], Chenxia Han[1], Dan Du[1,2]*

[1] *West China Centre of Excellence for Pancreatitis, Institute of Integrated Traditional Chinese and Western Medicine, West China Hospital/West China Medical School, Sichuan University, Chengdu 610041, China*

[2] *Advanced Mass Spectrometry Center, Research Core Facility, Frontiers Science Center for Disease-related Molecular Network, West China Hospital, Sichuan University, Chengdu 610041, China*

[3] *Peking Union Medical College Hospital, Chinese Academy of Medical Science & Peking Union Medical College, Beijing, 100730, China*

*Correspondence to: dudan1520@163.com

[#] These authors contributed equally to this manuscript.

Table S1. Histological scoring system for the evaluation of pancreatic injury in rats.

Table S2. Sequence of RT-qPCR primers.

Figure S1. Pancreatic and distal organ damage in the model of ornithine-induced severe acute pancreatitis (2.8 g/kg×2, 28%).

Figure S2. The differential lipids screened by time-course analysis and shown in a volcano plot.

Figure S3. The relative pancreatic mRNA expression of enzymes relevant to the lipid in Orn-SAP.

Table S1 Histological scoring system for the evaluation of pancreatic injury in rats.

	Score				
	0	1	2	3	4
Interstitial edema	0	Mild	Moderate	Severe	Extensive
Leukocyte adherence to vessel walls	0	Mild	Moderate	Extensive	-
Leukocyte infiltration	0	Focal	Diffuse/mild	Diffuse/moderate	Diffuse/severe
Vacuolization (% of total acinar cells)	0	Focal(＜10)	11-25	26-50	51-75
Necrosis (% of total acinar cells)	0	Focal(＜10)	11-25	26-50	51-75
Number of apoptotic bodies (% of total acinar cells)	0	＜5	6-10	11-20	-
Regeneration	0	Present	-	-	-

Table S2. Sequence of RT-qPCR primers.

**Oligonucleotide** (5'→3')	**Taget gene**	
CTA ACC TTG GGC AAA TCG AA	SPTC1	Forward
TGA GCA GGG AGA AGG GAC TA	SPTC1	Reverse
GGA CAG TGT GTG GCC TTT CT	SPTC2	Forward
TCA CTG AAG TGT GGC TCC TG	SPTC2	Reverse
CTC TGC TTC TCC TGG TTT GC	CERS2	Forward
CCA GCA GGT AGT CGG AAG AG	CERS2	Reverse
CGA GGC AGT TTC TGA AGG TC	CERS4	Forward
CCA TTG GTA ATG GCT GCT CT	CERS4	Reverse
CCAAAGTGGCTCCAGAAGAG	ACAT2	Forward
CCACACTGGCTTGTCGAGTA	ACAT2	Reverse
GAT GCT TGC TGT TCA CTC CA	GCS(UGCG)	Forward
GCT GAG ATG GAA GCC ATA GG	GCS(UGCG)	Reverse

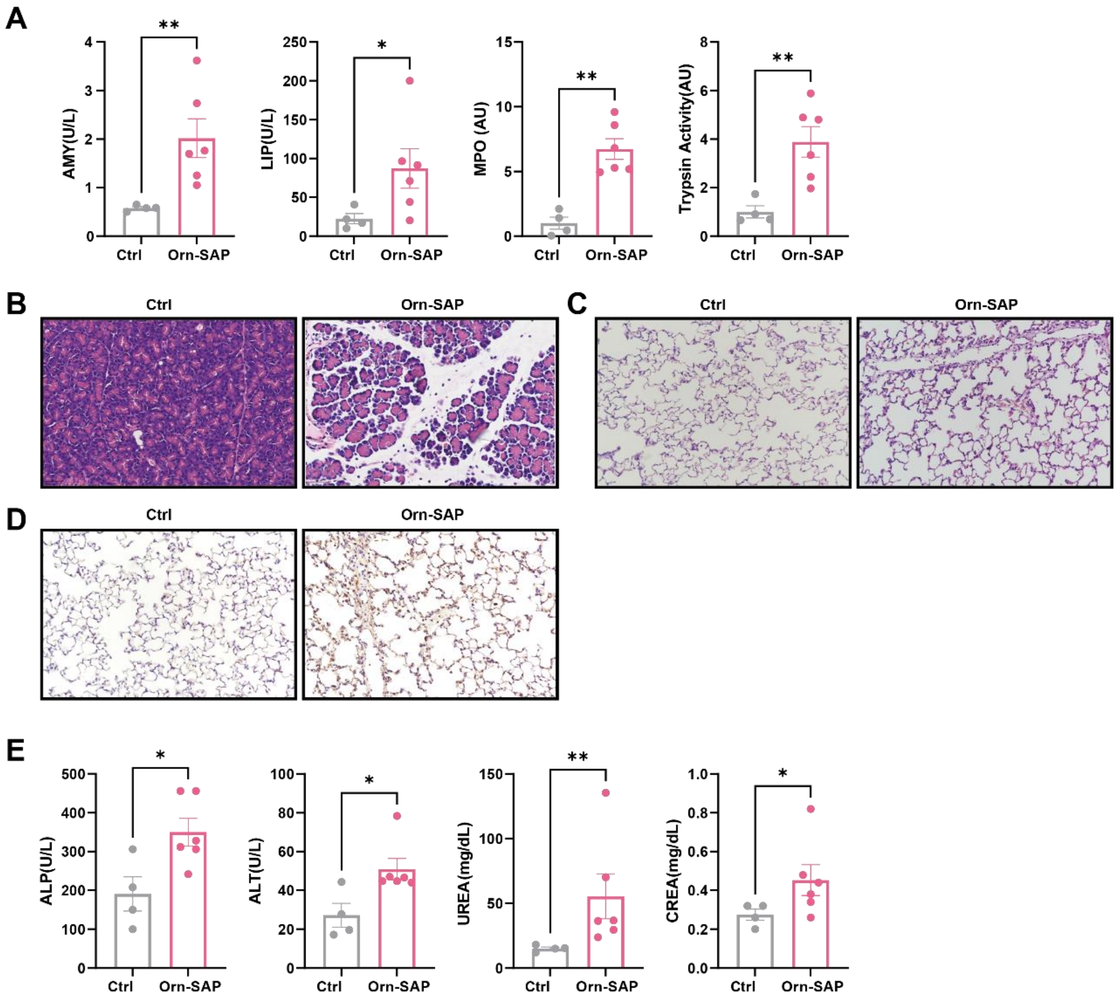

Figure S1. Pancreatic and distal organ damage in Orn-SAP. In the Orn-SAP group, acute pancreatitis was induced in rats through the administration of two intraperitoneal injections of L-ornithine (2.8g/kg,28%). The control (Ctrl) group received an equal volume of saline via the same route of administration. All animals were euthanized after 24 hours for subsequent analysis. (A) Indicators of pancreatitis including serum amylase activity, serum lipase activity, pancreatic myeloperoxidase activity, and pancreatic trypsin activity. (B) Representative H&E images of histopathologic changes in the pancreas (magnification 200×). (C) Representative H&E images of histopathologic changes in the lung (magnification 200×). (D) Representative immunohistochemistry images of MPO in pancreas sections (magnification 200×). (E) Hepatic impairment indicators, including serum alkaline phosphatase (ALP) and alanine transaminase (ALT) activity, as well as renal impairment indicators as serum urea and creatinine (CREA). $*P < 0.05$, $**P < 0.01$, and $***P < 0.001$ vs Ctrl; n = 4-6.

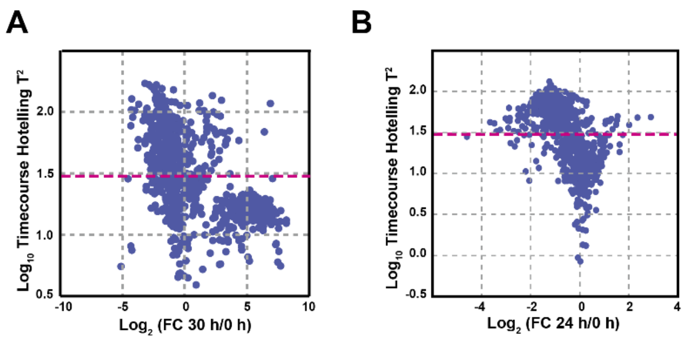

Figure S2. The differential lipids screened by time-course analysis and shown in a volcano plot. (A) It displayed the significant changed lipids in the pancreas along all timepoints by a time-course analysis with Hotelling T^2 score on Y-axis and fold change (FC) value on X-axis. (B) It displayed the significant changed lipids in the serum along all timepoints by a time-course analysis with Hotelling T^2 score on Y-axis and FC value on X-axis.

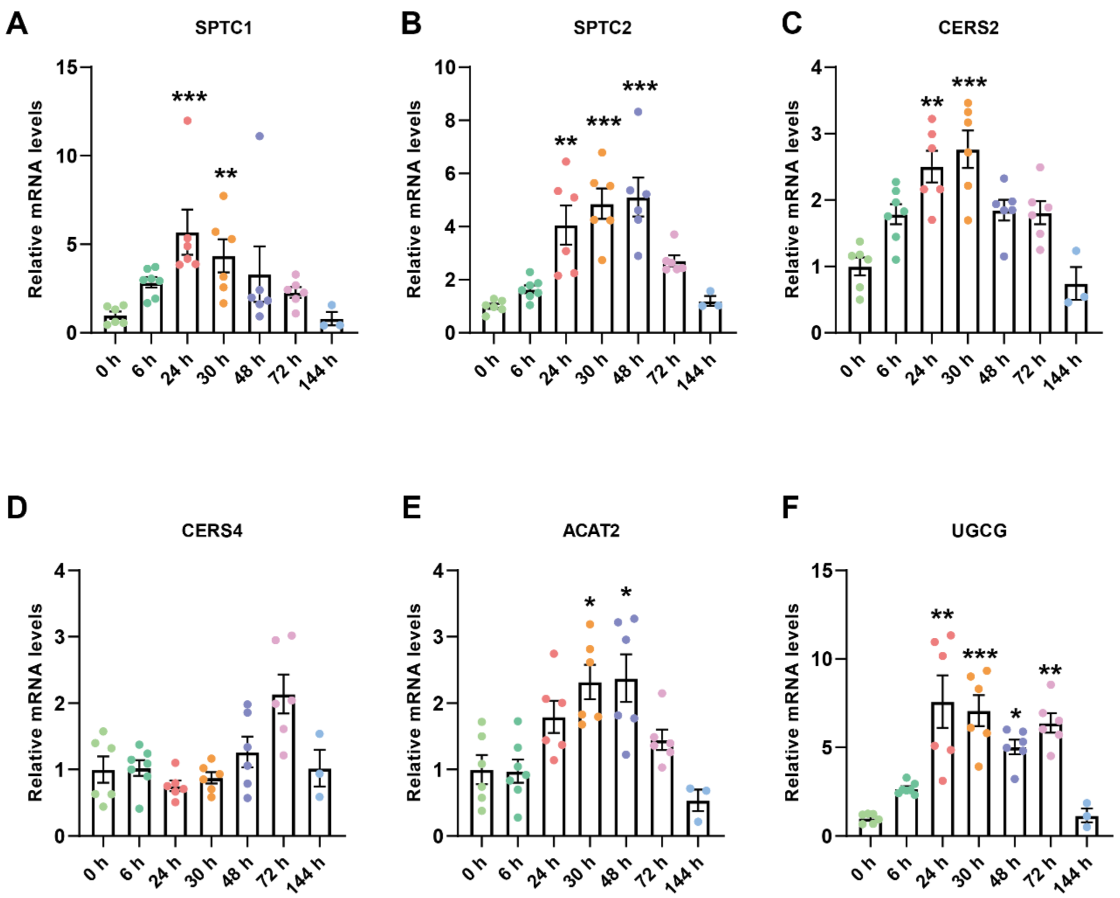

Figure S3. The relative pancreatic mRNA expression of enzymes relevant to the lipid in Orn-SAP. (A) Serine palmitoyltransferase long chain base subunit 1 (SPTLC1). (B) Serine palmitoyltransferase long chain base subunit 2 (SPTLC2). (C) Ceramide synthase 1 (CERS1). (D) Ceramide synthase 4 (CERS4). (E) acetyl-CoA acetyltransferase 2 (ACAT2). (F) UDP-glucose ceramide glucosyltransferase (GCS/UGCG). $*P < 0.05$, $**P < 0.01$, and $***P < 0.001$ vs 0 h; n = 6-8.
